# Cumulative Evidence for the Association of Thrombosis and the Prognosis of COVID-19: Systematic Review and Meta-Analysis

**DOI:** 10.3389/fcvm.2021.819318

**Published:** 2022-01-25

**Authors:** Dongqiong Xiao, Fajuan Tang, Lin Chen, Hu Gao, Xihong Li

**Affiliations:** ^1^Department of Emergency, West China Second University Hospital, Sichuan University, Chengdu, China; ^2^Key Laboratory of Birth Defects and Related Diseases of Women and Children (Sichuan University), Ministry of Education, Chengdu, China

**Keywords:** thrombosis and COVID-19 thrombosis, SARS-CoV-2, COVID-19, 2019-nCoV, mortality

## Abstract

**Background:**

Although thrombosis events have been reported in patients with coronavirus disease 2019 (COVID-19), the association between thrombosis and COVID-19-related critical status or risk of mortality in COVID-19 has been inconsistent.

**Objective:**

We conducted a meta-analysis of reports assessing the association between thrombosis and the prognosis of COVID-19.

**Methods:**

The EMBASE, Ovid-MEDLINE, and Web of Science databases were searched up to December 9, 2021, and additional studies were retrieved *via* manual searching. Studies were included if they reported the risk of COVID-19-related critical status or COVID-19-related mortality in relation to thrombosis. The related data were extracted by two authors independently, and a random effects model was conducted to pool the odds ratios (ORs). In addition, stratified analyses were conducted to evaluate the association.

**Results:**

Among 6,686 initially identified studies, we included 25 studies published in 2020 and 2021, with a total of 332,915 patients according to predefined inclusion criteria. The associations between thrombosis and COVID-19-related mortality and COVID-19-related critical status were significant, with ORs of 2.61 (95% CI, 1.91–3.55, *p* < 0.05) and 2.9 (95% CI, 1.6–5.24, *p* < 0.05), respectively. The results were statistically significant and consistent in stratified analyses.

**Conclusions:**

Thrombosis is associated with an increased risk of mortality and critical status induced by COVID-19. Further prospective studies with large sample sizes are required to establish whether these associations are causal by considering more confounders and to clarify their mechanisms.

Observational studies cannot prove causality. However, autopsy studies show thrombosis events preceding COVID-19-related deaths. The results of this meta-analysis reported that thrombosis was associated with a 161% increased risk of mortality from COVID-19 and a 190% increased risk of COVID-19-related critical status. The type of thrombosis included in the original studies also seemed to be related to the results.

## Introduction

Coronavirus disease 2019 (COVID-19), a novel infectious disease, is highly prevalent globally and has infected over 271 million patients to date (https://www.who.int/emergencies/diseases/novel-coronavirus-2019). COVID-19 is caused by severe acute respiratory syndrome coronavirus 2 (SARS-CoV-2), and progressive respiratory failure is the primary cause of death (1) during the COVID-19 pandemic. Over 5 million individuals globally have succumbed to COVID-19 (https://covid19.who.int/). However, little is known about the causes of death. Histologic autopsy of pulmonary vessels in patients with COVID-19 showed widespread thrombosis with microangiopathy (1–3). Luca Spiezia et al. (4) reported that severe hypercoagulability rather than consumptive coagulopathy station was observed in patients with COVID-19 with acute respiratory failure. Fibrin formation and polymerization may contribute to thrombosis and correlate with critical status and a worse outcome in patients with COVID-19 (4, 5). An increased risk of thrombosis, such as venous thromboembolism (VTE), brain stroke, cardiac ischemia, and pulmonary embolism (PE), in patients with COVID-19 admitted to the intensive care unit (ICU) has been reported (6–9). The magnitude of this public health challenge is increasing, a concerning trend given that COVID-19 imposes a significant public health burden and large demand on health care systems. The association between thrombosis and COVID-19 prognosis should be recognized by clinical doctors globally.

There were four types of thrombosis found in patients with COVID-19: pale thrombus, mixed thrombus (arterial and venous thrombosis), red thrombus, and hyaline thrombus (microvascular thrombosis). A hypercoagulable state in the critically ill patients with COVID-19 was found due to the following mechanisms: severe hypofibrinolysis (10), endothelial dysfunction (11, 12), platelet activation (12, 13), endothelial-derived von Willebrand factor (vWF) activation (14), elevated soluble (s) P-selectin (13, 15), gene expression (13, 16), inflammatory cytokine activation (17, 18), and mannose-binding lectin (MBL)-related complement activation (19, 20). Serious adverse events, such as thrombosis and thrombocytopenia syndrome, after COVID-19 vaccination are rare (21) and are associated with a high mortality rate (22). Campello et al. found that no hypercoagulable condition was found after COVID-19 (ChAdOx1 or BNT162b2) vaccination (23).

A number of primary studies (24–28) have evaluated the association between thrombosis and the risk of adverse outcomes of COVID-19, including mortality and severity of COVID-19, with inconsistent results. We, therefore, conducted a meta-analysis to evaluate the association between thrombosis and the prognosis of COVID-19.

## Methods

### Retrieval of Studies

The reporting of this meta-analysis of observational studies was in accordance with the Meta-Analysis of Observational Studies in Epidemiology (MOOSE) and Preferred Reporting Items for Systematic Reviews and Meta-Analyses (PRISMA) guidelines. The Embase, Ovid-MEDLINE, and Web of Science databases were searched up to 9 December 2021. The search consisted of three terms: thrombosis, COVID-19, and study design. We used the following key words to search for the first term: “thrombosis” OR “embolism” OR “thrombotic” OR “thrombus” OR “thrombi” OR “thromboembol^*^” OR “emboli^*^” OR “embolus” OR “clot?” OR “DVT” OR “VTE” OR “PE.” We used the following key words to search for the second term: “SARS-CoV-2” OR “COVID-19.” The third term was associated with “risk,” “mortality,” and “cohort.” Finally, we used “AND” to connect the three terms. For the search strategy, see Supplementary Material. The retrieved studies were first screened by reading the titles and abstracts. Two authors (Dongqiong Xiao and Hu Gao) independently read the full texts of the remaining studies. Fajuan Tang resolved any disagreements.

### Definition

The critical status among patients with COVID-19 is with any of the following conditions—shock, respiratory failure requiring mechanical ventilation, and/or other organ dysfunction requiring admission to the intensive care unit (ICU) (24).

### Study Selection

The inclusion criteria were as follows: (1) studies with participants who were investigated for the following outcomes: the incidence, prevalence, or risk or odds ratio (OR) of mortality and critical status in patients with COVID-19 with thrombosis relative to those without thrombosis; (2) studies that evaluated the association between thrombosis and prognosis of COVID-19 and reported unadjusted or adjusted ORs and their corresponding 95% confidence intervals (CIs) or the number of patients with COVID-19 with thrombosis relative to those without thrombosis; and (3) studies with case-control, cohort, or cross-sectional designs published in English.

The exclusion criteria were as follows: (1) studies that reported the results of few autopsy cases of COVID-19; (2) unrelated studies or studies in which the data overlapped with those of another study or studies that reported the association between the D-dimer level and COVID-19 without evidence of definite thrombosis; or (3) reviews, case reports, and meta-analyses.

### Data Extraction

The data were independently extracted from the studies by Dongqiong Xiao and Hu Gao, and they were aggregated in a standardized form; the collected data included study author and year, study location and design, sample size, type of thrombosis, primary outcomes (presence or absence of critical status, COVID-19-related mortality), adjusted for confounding factors, and Newcastle-Ottawa Scale (NOS) scores for the included studies.

### Quality Evaluation

The methodological quality of all the included studies (Supplementary Table 2) was examined by Dongqiong Xiao and Hu Gao independently using the NOS (29), and Fajuan Tang resolved any disagreements. The reviewers assessed the quality scores (varying from 0 to 9) in three domains: selection of the study population, evaluation of exposure and outcomes, and comparability.

### Statistical Analysis

The odds ratios (ORs) and 95% CIs were used as measures of the association between thrombosis and the prognosis of COVID-19 across studies. For original studies that compared the number of participants who developed critical status and death exposure to thrombosis compared with control groups, we calculated ORs and 95% CIs for each study (30). All data from the included studies were converted into log (ORs) and standard errors (SEs) (31). We pooled the log (ORs) and SEs of each study separately using the DerSimonian-Laird formula (random effects model) (32). We used the *I*^2^ statistic to assess the statistical heterogeneity among the studies (33). High heterogeneity was indicated with values of *I*^2^ > 50% and *p* < 0.05 (34).

We conducted stratified analyses based on the study location (Europe, the United States, and Asia), study design (cohort, cross-sectional), sample size (≥ 1,000 <1,000), type of thrombosis (VTE, PE, DVT, and others), adjusted for confounding factors [not available (NA), adjusted ≤ 7 factors, adjusted ≥ 8 factors, ≤ 7 factors], adjusted for age (yes, no), adjusted for sex (yes, no), adjusted for body mass index (BMI) (yes, no), adjusted for diabetes (yes, no), and adjusted for comorbidities (yes, no).

We used Egger's tests, Begg's tests, and funnel plots in the meta-analysis to assess publication bias (33–36). We used Stata software, version 12.0 (StataCorp, College Station, TX) and Review Manager, version 5.3 to perform the statistical tests.

## Results

### Literature Search

**Figure 1:**
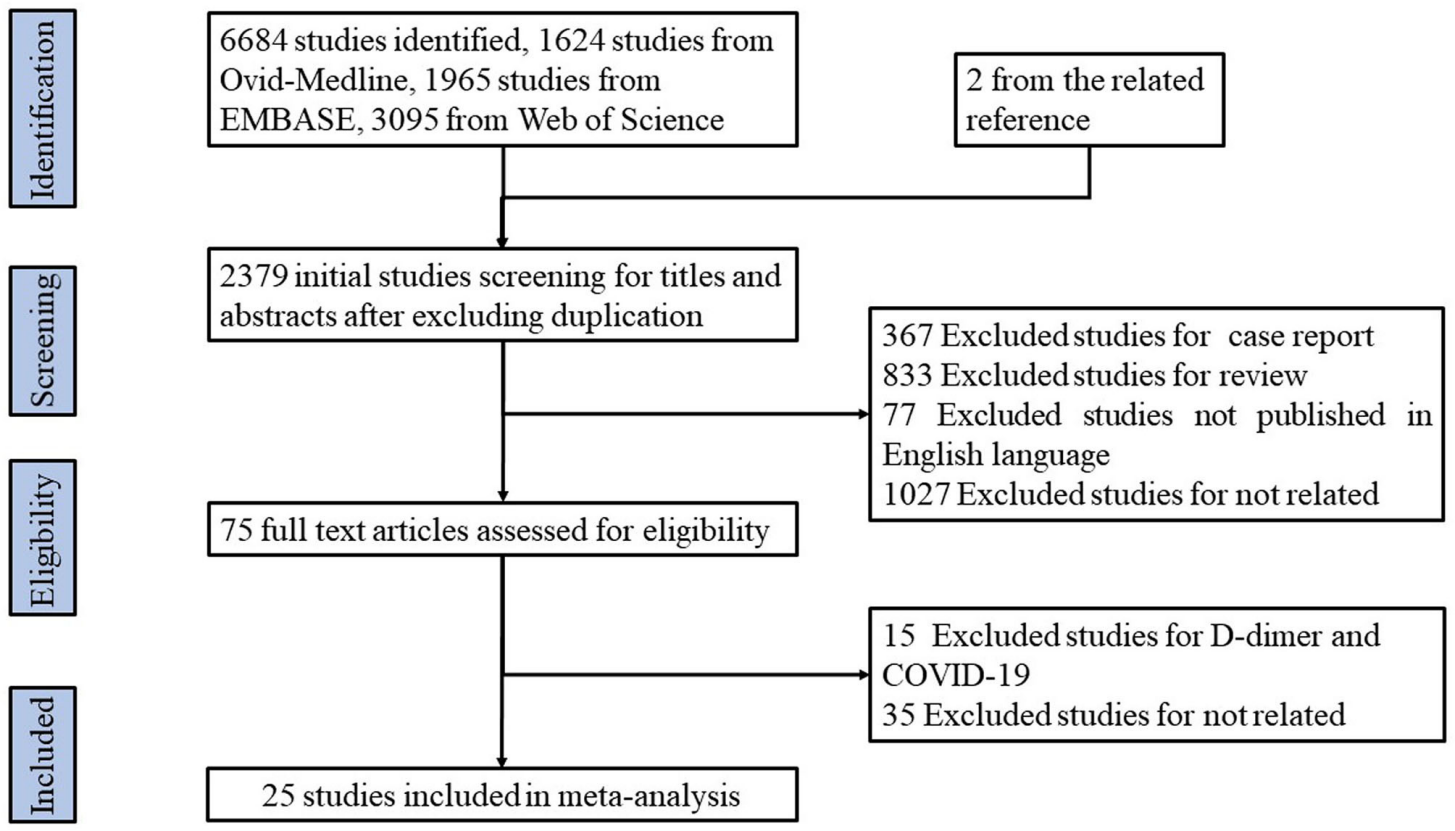
A flow chart describing study selection.

We identified 6,686 potential studies, including 1,624 from Ovid-MEDLINE, 1,965 from Embase, 3,095 from Web of Science, and 2 from the related references (Supplementary Table 3). After careful screening, 6,661 studies were excluded for the reasons listed in Figure 1, and 25 studies reporting the association between thrombosis and prognosis of COVID-19 met the inclusion criteria (see Figure 1). These 25 included studies are summarized in Table 1.

**Table 1:** Characteristics of the included studies.

**Study**	**Year**	**Study location**	**Sample size**	**Study design**	**Type of thrombosis**	**Outcomes**	**Adjusted for**
Zhang	2020	China	143	CSS	VTE	Mortality and critical care status	NA
Yaghi, Shadi	2020	United States	3,556	Retrospective cohort	Brain stroke	Mortality	Age and NIHSS score
Stoneham, Simon M.	2020	UK	230	CSS	VTE	ICU hospitalization	NA
Middeldorp, S.	2020	Netherlands	198	Retrospective cohort	VTE	Mortality and critical care status	Age, sex, and ICU stay
Leonard-Lorant, Ian	2020	France	106	Retrospective cohort	PE	ICU hospitalization	NA
Klok, F. A.	2020	Netherlands	184	Retrospective cohort	Thrombotic complications	Mortality	NA
Jain, R.	2020	United States	3,218	Retrospective cohort	Brain stroke	Mortality	Age, BMI, and hypertension
Bhayana, R.	2020	United States	412	CSS	Abdominal ischaemia	ICU hospitalization	NA
Ren, B.	2020	China	48	CSS	VTE	Mortality	NA
Galloway, James B	2020	UK	1,157	Retrospective cohort	Cardiac ischaemia	Mortality and critical care status	>8 factors, age, sex, and with comorbidities (such as hypertension and diabetes mellitus)
Corrado Lodigiani	2020	Italy	338	Retrospective cohort	VTE	ICU hospitalization	NA
Avruscio	2020	Italy	85	Observational cohort	VTE	ICU hospitalization	NA
Contou	2020	France	92	CSS	PE	Mortality	NA
Abizaid	2021	Brazil	152	Prospective study	MI	Mortality	Age, prior coronary disease, and myocardial blush
Alharthy	2021	Saudi Arabia	352	Retrospective study	PE	Mortality	Age, ICU length of stay, SpO_2_/FiO_2_ ratio, WBCs, lymphocytes, D-dimer, lactate, and active smoking
Alwafi	2021	Saudi Arabia	706	CSS	VTE	Mortality	Age, sex, and comorbidities (diabetes mellitus, hypertension, coronary artery disease, end-stage renal disease, asthma, congestive heart failure, cerebrovascular accident, chronic obstructive pulmonary disease, chronic liver disease, and cancer)
Anderson	2021	UK	312,378	Cohort	VTE	Mortality Critical status	Comorbid cardiovascular disease (myocardial infarction, heart failure, angina, stroke, transient ischaemic attack, atrial fibrillation/flutter, and valve disease) and prevalent diabetes mellitus; use of exogenous oestrogens in women only
Arribalzaga	2021	Spain	5,966	Cohort	VTE	Mortality	Age, sex, follow-up (days), and time from admission to VTE diagnosis
Fournier	2021	France	531	Cohort	Arterial thrombotic events	Mortality	Age, sex, and comorbidities (cancer, HIV infection, inflammatory disorders, high blood pressure, smoking, and diabetes)
Purroy	2021	Spain	1,737	Cohort	Thromboem- bolism	Mortality	Age, diabetes, chronic obstructive pulmonary disease, ICU care, systolic blood pressure, and oxygen saturation
Riyahi	2021	USA	413	Retrospective cohort	PE	Mortality	NA
Scudiero	2021	Italy	224	Retrospective cohort	PE	Mortality	Age, sex, and comorbidities
Violi	2021	Italy	373	Prospective multicentre study	Thrombotic events	Mortality	Age, sex, COPD, diabetes, and D-dimer
Wang	2021	China	88	Retrospective	DVT	Critical status	NA
Paz Rios	2021	USA	184	Retrospective observational study	VTE	Mortality	Age, sex, race, comorbidities (diabetes, hypertension, COPD, CKD, heart failure, cancer, and atrial fibrillation)

*CSS, cross-sectional study; COPD, chronic obstructive pulmonary disease; CKD, chronic kidney disease; DVT, deep venous thrombosis; HIV, human immunodeficiency virus; ICU, intensive care unit; MI, myocardial infarction; NA, not available; PE, pulmonary embolism; USA, United States of America; VTE, venous thromboembolism; WBC, white blood cell*.

### Characteristics and Quality of the Included Studies

Table 1 shows the characteristics of the 25 included studies. Among the included studies, 6 studies (24, 26, 37–40) were cross-sectional studies, and 19 studies (7, 25, 27, 28, 41–55) were cohort studies. The association between thrombosis and COVID-19-related mortality was the primary outcome of interest in 19 studies, and the association between thrombosis and COVID-19-related critical status was the primary outcome in 10 studies.

The related studies were published in 2020 and 2021, and the sample size ranged from 48 to 312,378, for a total of 332,915 participants across studies.

Five studies (25, 38, 42, 51, 55) were conducted in the United States, 5 studies (24, 26, 39, 46, 54) were conducted in Asia, 14 studies (7, 27, 28, 37, 40, 41, 43, 44, 47–50, 52, 53) were conducted in Europe, and one study (45) was conducted in Brazil. All the included studies included both adult men and women.

Among the included studies, 13 studies (25–27, 39, 42, 45, 46, 48–50, 52, 53, 55) adjusted for age, 7 studies (27, 39, 48, 49, 52, 53, 55) adjusted for sex, one study (42) adjusted for BMI, 8 studies (26, 39, 47, 49, 50, 52, 53, 55) adjusted for diabetes mellitus, and 7 studies (39, 43, 46, 47, 49, 52, 55) adjusted for 8 or more confounding factors.

The quality scores of the included studies ranged from 6 to 8 (Supplementary Table 1), and they were considered high.

### Quantitative Results (Meta-Analysis)

**Figure 2:**
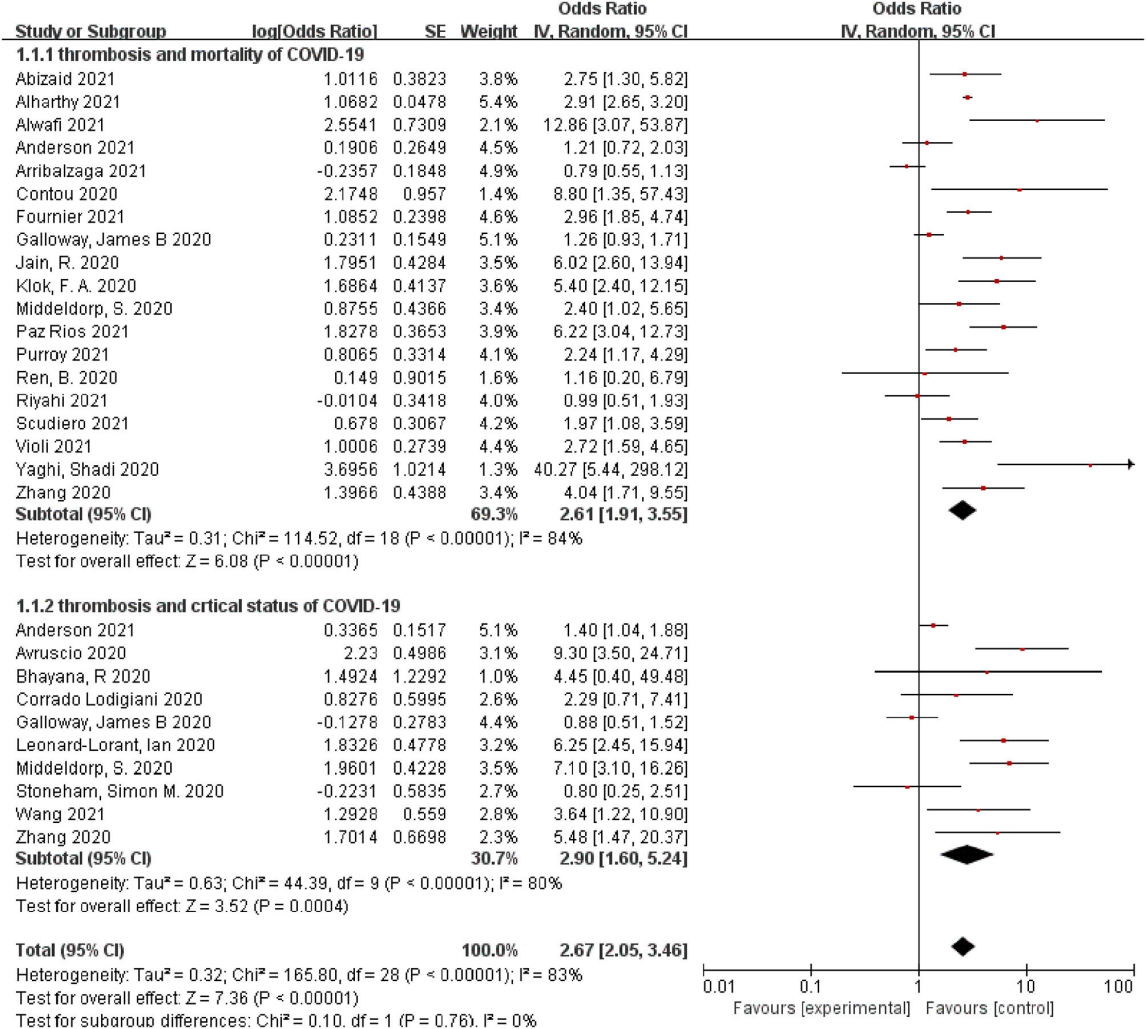
A forest plot of the pooled odds ratio of the association between thrombosis and prognosis of COVID-19, including mortality and critical status.

Among the 25 selected studies, 19 studies revealed the association between thrombosis and COVID-19-related mortality, and 10 studies investigated the association between thrombosis and COVID-19-related critical status. Among the included studies, 5 studies (26, 43, 47, 48, 51) found a non-significant association between thrombosis and COVID-19-related mortality, while the other 14 studies (24, 25, 27, 28, 39, 40, 42, 45, 46, 49, 50, 52, 53, 55) revealed that thrombosis would increase the risk of mortality from COVID-19. All 19 studies reported risks as ORs, ranging from 0.79 to 40.27. Any type of thrombosis was associated with an increased risk of mortality from COVID-19 compared with the control, with a pooled OR of 2.61 (95% CI, 1.91, 3.55). High heterogeneity was found in these studies (*I*^2^ = 84%, *p* < 0.05) (Figure 2).

Additionally, among the included studies, 4 studies (7, 37, 38, 43) found a non-significant association between thrombosis and COVID-19-related critical status, while the other 6 studies (24, 27, 41, 44, 47, 54) revealed that thrombosis would increase the risk of COVID-19-related critical status. All seven studies reported risks as ORs, ranging from 0.8 to 9.3. Any type of thrombosis was associated with an increased risk of COVID-19-related critical status compared with the control, with a pooled OR of 2.9 (95% CI, 1.6, 5.24). High heterogeneity was reported in the studies (*I*^2^ = 80%, *p* < 0.05) (Figure 2).

### Stratified Analyses

#### Thrombosis and COVID-19-Related Mortality

Among the 25 selected studies, 19 studies revealed the association between thrombosis and COVID-19-related mortality. Stratified analyses of clinical factors and study characteristics were conducted to evaluate possible sources of heterogeneity in the included studies (Table 2). The association between thrombosis and COVID-19-related mortality was significant at 2.61 (95% CI, 1.91, 3.55), and this association was consistent in all of the stratified analyses (Table 2). Stronger associations between thrombosis and the COVID-19-related mortality were found in cross-sectional studies (OR: 4.86, 95% CI, 1.99, 11.83) when compared to that in cohort studies (OR: 2.39, 95% CI, 1.72, 3.33) in studies with small sample sizes (<1,000) (OR: 2.95, 95% CI, 2.28, 3.82) when compared to studies with large sample sizes (≥ 1,000) (OR: 1.99, 95% CI, 1.1, 3.58), and in studies that were conducted in the United States compared with studies conducted in Europe and Asia (Table 2).

**Table 2:** Stratified analysis of the associations between thrombosis and mortality and COVID-19-related critical status.

	**Thrombosis and mortality**				**Thrombosis and critical status**			
**Variables**	**Studies**	**OR (95% CI)**	***I^**2**^* (*P-value*)**	***P***	**Studies**	**OR (95% CI)**	***I^**2**^* (*P-value*)**	***P***
Total	19	2.61 (1.91, 3.55)	84% (<0.05)		10	2.9 (1.6, 5.24)	83% (<0.05)	
**Study location**								
Europe	10	2.01 (1.37, 2.95)	79% (<0.05)	**<0.05**	7	2.58 (1.28, 5.19)	85% (<0.05)	**<0.05**
Unites States-Brazil	5	4.24 (1.67, 10.76)	83% (<0.05)		1	4.45 (0.4, 49.48)	NA	
Asia	4	3.51 (1.95, 6.3)	47% (0.13)		2	4.31 (1.86, 9.99)	0 (0.64)	
**Study design**								
Cohort	15	2.39 (1.72, 3.33)	87% (<0.05)	**<0.05**	7	3.11 (0.55, 6.2)	85% (<0.05)	*>0.05*
Cross-sectional	4	4.86 (1.99, 11.83)	35% (0.18)		3	2.38 (0.58, 9.76)	61% (0.08)	
**Sample size**								
≥1,000	6	1.99 (1.1, 3.58)	85% (<0.05)	*>0.05*	2	1.18 (0.76, 1.83)	53% (0.14)	**<0.05**
<1,000	13	2.95 (2.28, 3.82)	53% (0.01)		8	4.17 (2.37, 7.35)	50% (0.05)	
**Type of thrombosis**								
VTE	7	2.48 (1.17, 5.25)	86% (<0.05)	**<0.05**	6	2.67 (1.28, 5.59)	75% (<0.05)	**<0.05**
PE	4	2.16 (1.18, 3.93)	76% (<0.05)		1	6.25 (2.45, 15.94)	NA	
DVT	0	NA	NA		1	3.64 (1.22, 10.90)	NA	
Other	8	3.17 (1.95, 5.16)	79% (<0.05)		2	1.27 (0.34, 4.38)	39%(0.2)	
**Adjusted for confounding factors**								
NA	5	2.81 (1.16, 6.78)	72% (<0.05)	**<0.05**	7	3.74 (1.95, 7.16)	52% (0.05)	**<0.05**
Adjusted (≤7 factors)	6	3.06 (1.35, 6.95)	88% (<0.05)		1	7.1 (3.1, 16.26)	NA	
Adjusted (≥8 factors)	8	2.25 (1.54, 3.31)	86% (<0.05)		2	1.18 (0.76, 1.83)	53% (0.14)	
**Adjusted for age**								
Yes	12	2.8 (1.91, 4.1)	88% (<0.05)	***>**0.05*	2	2.44 (0.32, 18.87)	94% (<0.05)	*>0.05*
No	7	2.29 (1.26, 4.17)	68% (<0.05)		8	3.1 (1.59, 6.06)	74% (<0.05)	
**Adjusted for sex**								
Yes	8	2.39 (1.43, 3.97)	87% (<0.05)	*>0.05*	2	2.44 (0.32, 18.87)	94% (<0.05)	*>0.05*
No	11	2.84 (1.92, 4.18)	72% (<0.05)		8	3.1 (1.59, 6.06)	74% (<0.05)	
**Adjusted for BMI**								
Yes	1	6.02 (2.6, 13.64)	NA	**<0.05**	0	NA	NA	NA
No	18	2.49 (1.82, 3.42)	85% (<0.05)		10	2.9 (1.6, 5.24)	83% (<0.05)	
**Adjusted for diabetes**								
Yes	7	2.59 (1.56, 4.31)	81% (<0.05)	*>0.05*	2	1.18 (0.76, 1.83)	53% (0.14)	**<0.05**
No	12	2.69 (1.74, 4.16)	81% (<0.05)		8	4.17 (2.37, 7.35)	78% (<0.05)	
**Adjusted for comorbidities**								
yes	6	2.53 (1.44, 4.44)	84% (<0.05)	*>0.05*	2	1.18 (0.76, 1.83)	53% (0.14)	**<0.05**
no	13	2.71 (1.81,4.07)	83% (<0.05)		8	4.17 (2.37, 7.35)	78% (<0.05)	

*BMI, body mass index; DVT, deep venous thrombosis; NA, not available; PE, pulmonary embolism; VTE, venous thromboembolism. Significantly different (p < 0.05)*.

The type of thrombosis included in the original reports also seemed to be related to the results. For example, studies demonstrated a weaker association between thrombosis and the COVID-19-related mortality if the thrombosis was VTE (OR: 2.48, 95% CI, 1.17, 5.25) when compared to other types of thrombosis (OR: 3.17, 95% CI, 1.95, 5.16).

The association between thrombosis and the COVID-19-related mortality was strong when the studies were not adjusted for sex, diabetes, comorbidities, or <8 confounding factors (Table 2).

#### Thrombosis and COVID-19-Related Critical Status

Among the 25 selected studies, 10 studies investigated the association between thrombosis and COVID-19-related critical status. The same stratified analyses were conducted (Table 2). The association between thrombosis and COVID-19-related critical status was significant (OR: 2.9, 95% CI, 1.6, 5.24), and it was consistent in all of the stratified analyses (Table 2). Sample size, study location, type of thrombosis, adjusted for more than 8 confounding factors, diabetes, and comorbidities seemed to be correlated with the results. For example, stronger associations between thrombosis and COVID-19-related critical status were found in studies that were conducted in Asia (OR: 4.31, 95% CI, 1.86, 9.99) when compared to those in studies that were conducted in Europe (OR: 2.58, 95% CI, 1.28, 5.19) and in studies with a small sample size (<1,000) (OR: 4.17, 95% CI, 2.37, 7.35) when compared to those in studies with a large sample size (≥ 1,000) (OR: 1.18, 95% CI, 0.76, 1.83) (Table 2).

The association between thrombosis and COVID-19-related critical status was strong when the studies were not adjusted for diabetes, comorbidities, or <8 confounding factors (Table 2).

### Publication Bias

**Figure 3:**
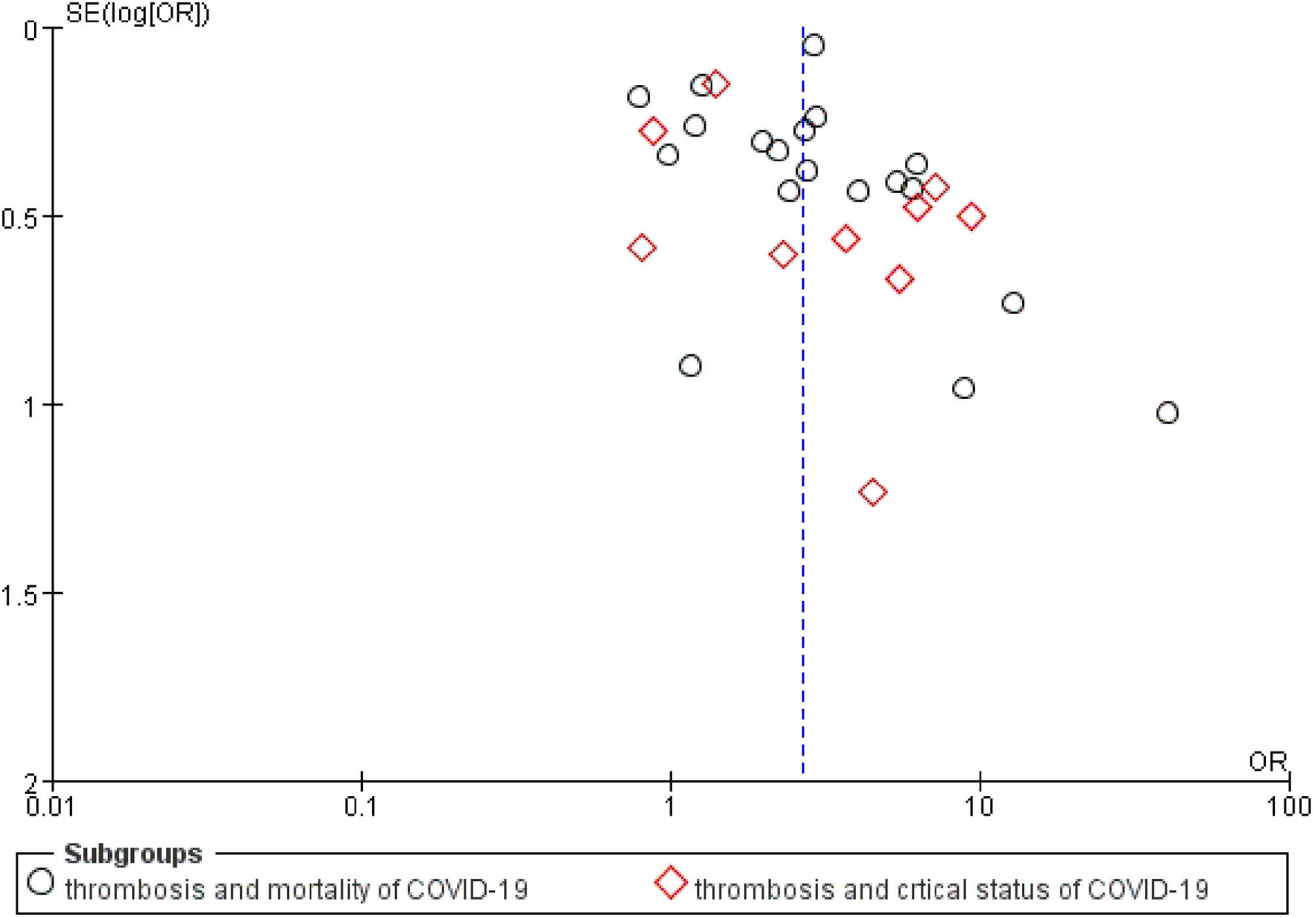
A funnel plot of public bias of the association between thrombosis and prognosis of COVID-19.

Potential publication bias was revealed by asymmetrical funnel plots (Figure 3). The publication bias test for the association between thrombosis and COVID-19-related mortality was not significant (Begg's test with *p* = 0.069, *z* = 1.82), and publication bias was also not statistically significant for the association between thrombosis and COVID-19-related critical status with Begg's test (*p* = 0.858, *z* = 0.18) (Supplementary Table 4).

## Discussion

To the best of our knowledge, this study tried to evaluate the association between thrombosis and the prognosis of COVID-19, which is often neglected by clinical physicians. The results of this meta-analysis, which included 25 studies, revealed that thrombosis was associated with a 161 and 190% increased risk of COVID-19-related mortality and COVID-19-related critical status, respectively. The association persisted and remained statistically significant in all of the stratified analyses.

Observational studies cannot prove causality. However, the following issues may explain the causation. First, there was an appropriate temporal relationship: thrombosis preceded COVID-19-related mortality in all studies. Second, there is theoretical biological plausibility for causality in that thrombosis may lead to organ dysfunction or prolong hypoxia, critical status, and death. The high rate of death-causing pulmonary embolism at autopsy is one of the strongest prognostic markers of a poor outcome (2). Additionally, the lungs of patients with COVID-19 displayed severe endothelial injury and diffuse thrombosis with microangiopathy (1, 56, 57). The association between deep venous thrombosis (DVT) and COVID-19 is uncertain, and the mechanisms may be related to the following factors: the coagulation system may be activated by SARS-CoV-2, viral infection-induced release of cytokine, which is also thrombogenic, the plausible role of angiotensin-converting enzyme receptors induced severe endothelial injury, a pro-coagulatory state by tissue factor pathway activation (2, 4, 8, 58). Third, the findings revealed stronger associations for other thromboses, such as brain stroke and PE, relative to VTE. Hypoxia of important organs may lead to critical status and death (59). Fourth, there was consistency of this association across the included studies, as shown by the forest plot (Figure 2).

Conversely, there are also possible non-causal explanations for this association. Thrombosis is often associated with other confounding factors, including lack of physical activity, obesity, diabetes, hypertension, older age, sex, and chronic organ diseases (60, 61). Some of these factors were adjusted for the studies included in our meta-analysis, but the extent to which these potential intervening factors were controlled for in the individual studies was generally limited. The lack of adjustment for age (only 13 studies adjusted for age), sex (only 9 studies), BMI, diabetes, and comorbidities (only 7 studies) could contribute to a non-causal association between thrombosis and the COVID-19-related critical status and COVID-19-related mortality.

Our meta-analysis reports a stronger association between thrombosis and mortality without adjusting for sex relative to adjusting for sex. In our meta-analysis, two studies reported an association adjusted for sex. Xie et al. (62) may explain that age and sex are related to the COVID-19-related mortality. The authors reported that ACE2 concentration decreased almost 67% in older female rats and 78% in older male rats relative to younger groups. Additionally, evidence shows that sex hormones may modulate the expression of ACE2 (63). Kuba et al. (64) identified that ACE2 protects against acute lung injury, and decreased ACE2 may be related to the adverse outcome of COVID-19. The risk of severe infection and mortality increase with male sex (65). Sex was a strong factor in the COVID-19-related mortality, and several studies support this result (66, 67).

Our meta-analysis has many limitations. First, the sample size of the included studies was small, and the results of this meta-analysis should be interpreted with caution. Second, some of the included studies reported the association among thrombosis and mortality and critical status without adjustment for confounding factors, such as crude ORs or number of participants, which may have led to high heterogeneity and an overestimation of the results of the meta-analysis. Third, some related studies may be omitted by the study selection. Fourth, potential publication bias existed because studies published in English and articles were included. Fifth, there was no analysis of the association between different types of thrombosis and different statuses of COVID-19 based on the original studies. Furthermore, quantitative synthesis could not eliminate the bias inherent to observational studies.

There are a few merits of this meta-analysis. First, this study evaluated the association among thrombosis and mortality and the COVID-19-related critical status globally. Considering the consistent finding of increased mortality and critical status associated with thrombosis, we recommend that further prospective cohort studies considering additional adjusted confounding factors should be performed to test this hypothesis. Second, this study demonstrated that study location, study design, sample size, type of thrombosis, and adjusted confounding factors were all sources of heterogeneity.

## Conclusions

In conclusion, our pooled analyses provide evidence that participants with thrombosis were associated with an increased risk of COVID-19-related mortality and COVID-19-related critical status. Further prospective studies with large sample sizes are required to establish whether this association is causal by considering more confounders and to clarify its mechanisms.

## Data Availability Statement

The original contributions presented in the study are included in the article/Supplementary Material, further inquiries can be directed to the corresponding author/s.

## Author Contributions

DX, HG, and FT: conceptualization. HG, FT, LC, and DX: methodology. DX, HG, FT, LC, and XL: software, validation, formal analysis, investigation, resources, data curation, and visualization. DX and FT: writing—original draft preparation. DX and XL: writing—review and editing and supervision. All authors read and approved the final manuscript.

## Funding

The present study was supported by the National Science Foundation of China (Grant Nos. 82001593 and 82071353). It was supported by the Key R&D Projects of Science and Technology Department of Sichuan Province (Grant Nos. 2021YFS0029 and 2020YFS104).

## Conflict of Interest

The authors declare that the research was conducted in the absence of any commercial or financial relationships that could be construed as a potential conflict of interest.

## Publisher's Note

All claims expressed in this article are solely those of the authors and do not necessarily represent those of their affiliated organizations, or those of the publisher, the editors and the reviewers. Any product that may be evaluated in this article, or claim that may be made by its manufacturer, is not guaranteed or endorsed by the publisher.

## Abbreviations

BMI: body mass index
COVID-19: coronavirus disease 2019
DVT: deep venous thrombosis
ICU: intensive care unit
PE: pulmonary embolism
SARS-CoV-2: severe acute respiratory syndrome coronavirus 2
VTE: venous thromboembolism.

## References

[1] Ackermann M Verleden SE Kuehnel M Haverich A Welte T Laenger F . Pulmonary vascular endothelialitis, thrombosis, and angiogenesis in Covid-19. N Engl J Med. (2020) 383:120–8. 10.1056/NEJMoa2015432 32437596 PMC7412750

[2] Wichmann D Sperhake J-P Lutgehetmann M Steurer S Edler C Heinemann A . Autopsy findings and venous thromboembolism in patients with COVID-19. Ann Intern Med. (2020) 173:268–77. 10.7326/M20-2003 32374815 PMC7240772

[3] Ciceri F Beretta L Scandroglio AM Colombo S Landoni G Ruggeri A . Microvascular COVID-19 lung vessels obstructive thromboinflammatory syndrome (MicroCLOTS): an atypical acute respiratory distress syndrome working hypothesis. Crit Care Resusc. (2020) 22:95–7. 10.51893/2020.2.pov2 32294809 PMC10692450

[4] Spiezia L Boscolo A Poletto F Cerruti L Tiberio I Campello E . COVID-19-related severe hypercoagulability in patients admitted to intensive care unit for acute respiratory failure. Thromb Haemost. (2020) 120:998–1000. 10.1055/s-0040-1710018 32316063 PMC7295272

[5] Spiezia L Campello E Cola M Poletto F Cerruti L Poretto A . More severe hypercoagulable state in acute COVID-19 pneumonia as compared with other pneumonia. Mayo Clin Proc Innov Qual Outcomes. (2020) 4:696–702. 10.1016/j.mayocpiqo.2020.09.002 33024937 PMC7528900

[6] Demelo-Rodriguez P Cervilla-Munoz E Ordieres-Ortega L Parra-Virto A Toledano-Macias M Toledo-Samaniego N . Incidence of asymptomatic deep vein thrombosis in patients with COVID-19 pneumonia and elevated D-dimer levels. Thromb Res. (2020) 192:23–6. 10.1016/j.thromres.2020.05.018 32405101 PMC7219400

[7] Lodigiani C Iapichino G Carenzo L Cecconi M Ferrazzi P Sebastian T . Venous and arterial thromboembolic complications in COVID-19 patients admitted to an academic hospital in Milan, Italy. Thromb Res. (2020) 191:9–14. 10.1016/j.thromres.2020.04.024 32353746 PMC7177070

[8] Thomas W Varley J Johnston A Symington E Robinson M Sheares K . Thrombotic complications of patients admitted to intensive care with COVID-19 at a teaching hospital in the United Kingdom. Thromb Res. (2020) 191:76–7. 10.1016/j.thromres.2020.04.028 32402996 PMC7182517

[9] Llitjos J-F Leclerc M Chochois C Monsallier J-M Ramakers M Auvray M . High incidence of venous thromboembolic events in anticoagulated severe COVID-19 patients. J Thromb Haemost. (2020) 18:1743–6. 10.1111/jth.14869 32320517 PMC7264774

[10] Kruse JM Magomedov A Kurreck A Münch FH Koerner R Kamhieh-Milz J . Thromboembolic complications in critically ill COVID-19 patients are associated with impaired fibrinolysis. Crit Care. (2020) 24:676. 10.1186/s13054-020-03401-8 33287877 PMC7719734

[11] Aid M Busman-Sahay K Vidal SJ Maliga Z Bondoc S Starke C . Vascular disease and thrombosis in SARS-CoV-2-infected rhesus macaques. Cell. (2020) 183:1354–66.e13. 10.1016/j.cell.2020.10.005 33065030 PMC7546181

[12] Goshua G Pine AB Meizlish ML Chang CH Zhang H Bahel P . Endotheliopathy in COVID-19-associated coagulopathy: evidence from a single-centre, cross-sectional study. Lancet Haematol. (2020) 7:e575–82. 10.1016/S2352-3026(20)30216-7 32619411 PMC7326446

[13] Yatim N Boussier J Chocron R Hadjadj J Philippe A Gendron N . Platelet activation in critically ill COVID-19 patients. Ann Intensive Care. (2021) 11:113. 10.1186/s13613-021-00899-1 34273008 PMC8286043

[14] Mei ZW van Wijk XMR Pham HP Marin MJ. Role of von Willebrand factor in COVID-19 associated coagulopathy. J Appl Lab Med. (2021) 6:1305–15. 10.1093/jalm/jfab042 33930144 PMC8135722

[15] Agrati C Bordoni V Sacchi A Petrosillo N Nicastri E Del Nonno F . Elevated P-Selectin in severe Covid-19: considerations for therapeutic options. Mediterr J Hematol Infect Dis. (2021) 13:e2021016. 10.4084/mjhid.2021.016 33747397 PMC7938922

[16] Calabrese C Annunziata A Coppola A Pafundi PC Guarino S Di Spirito V . ACE Gene I/D polymorphism and acute pulmonary embolism in COVID19 pneumonia: a potential predisposing role. Front Med. (2020) 7:631148. 10.3389/fmed.2020.631148 33585520 PMC7874110

[17] Chen Y Wang J Liu C Su L Zhang D Fan J . IP-10 and MCP-1 as biomarkers associated with disease severity of COVID-19. Mol Med. (2020) 26:97. 10.1186/s10020-020-00230-x 33121429 PMC7594996

[18] Conti P Caraffa A Gallenga CE Ross R Kritas SK Frydas I . IL-1 induces throboxane-A2 (TxA2) in COVID-19 causing inflammation and micro-thrombi: inhibitory effect of the IL-1 receptor antagonist (IL-1Ra). J Biol Regul Homeost Agents. (2020) 34:1623–7. 10.23812/20-34-4EDIT-65 32744052

[19] Ma L Sahu SK Cano M Kuppuswamy V Bajwa J McPhatter J . Increased complement activation is a distinctive feature of severe SARS-CoV-2 infection. Sci Immunol. (2021) 6:eabh2259. 10.1126/sciimmunol.abh2259 34446527 PMC8158979

[20] Eriksson O Hultström M Persson B Lipcsey M Ekdahl KN Nilsson B . Mannose-binding lectin is associated with thrombosis and coagulopathy in critically ill COVID-19 patients. Thromb Haemost. (2020) 120:1720–4. 10.1055/s-0040-1715835 32871607 PMC7869044

[21] Taquet M Husain M Geddes JR Luciano S Harrison PJ. Cerebral venous thrombosis and portal vein thrombosis: a retrospective cohort study of 537,913 COVID-19 cases. EClinicalMedicine. (2021) 39:101061. 10.1016/j.eclinm.2021.101061 34368663 PMC8324974

[22] Wiedmann M Skattør T Stray-Pedersen A Romundstad L Antal EA Marthinsen PB . Vaccine induced immune thrombotic thrombocytopenia causing a severe form of cerebral venous thrombosis with high fatality rate: a case series. Front Neurol. (2021) 12:721146. 10.3389/fneur.2021.721146 34393988 PMC8363077

[23] Campello E Simion C Bulato C Radu CM Gavasso S Sartorello F . Absence of hypercoagulability after nCoV-19 vaccination: an observational pilot study. Thromb Res. (2021) 205:24–8. 10.1016/j.thromres.2021.06.016 34246010 PMC8231699

[24] Zhang L Feng X Zhang D Jiang C Mei H Wang J . Deep vein thrombosis in hospitalized patients with coronavirus disease 2019 (COVID-19) in Wuhan, China: prevalence, risk factors, and outcome. Circulation. (2020) 142:114–28. 10.1161/CIRCULATIONAHA.120.046702 32421381

[25] Yaghi S Ishida K Torres J Mac Grory B Raz E Humbert K . SARS2-CoV-2 and stroke in a New York healthcare system. Stroke. (2020) 51:2002–11. 10.1161/STROKEAHA.120.030335 32432996 PMC7258764

[26] Ren B Yan F Deng Z Zhang S Xiao L Wu M . Extremely high incidence of lower extremity deep venous thrombosis in 48 patients with severe COVID-19 in Wuhan. Circulation. (2020) 142:181–3. 10.1161/CIRCULATIONAHA.120.047407 32412320

[27] Middeldorp S Coppens M van Haaps TF Foppen M Vlaar AP Müller MCA . Incidence of venous thromboembolism in hospitalized patients with COVID-19. J Thromb Haemost. (2020) 18:1995–2002. 10.20944/preprints202004.0345.v1 32369666 PMC7497052

[28] Klok FA Kruip MJHA van der Meer NJM Arbous MS Gommers D Kant KM . Confirmation of the high cumulative incidence of thrombotic complications in critically ill ICU patients with COVID-19: An updated analysis. Thromb Res. (2020) 191:148–50. 10.1016/j.thromres.2020.04.041 32381264 PMC7192101

[29] Gou X Pan L Tang F Gao H Xiao D. The association between vitamin D status and tuberculosis in children: a meta-analysis. Medicine. (2018) 97:e12179. 10.1097/MD.0000000000012179 30170465 PMC6392646

[30] Xiao D Zhang X Ying J Zhou Y Li X Mu D . Association between vitamin D status and sepsis in children: a meta-analysis of observational studies. Clin Nutr. (2019) 39:1735–41. 10.1016/j.clnu.2019.08.010 31495735

[31] Willi C Bodenmann P Ghali WA Faris PD Cornuz J. Active smoking and the risk of type 2 diabetes: a systematic review and meta-analysis. JAMA. (2007) 298:2654–64. 10.1001/jama.298.22.2654 18073361

[32] Hartzel J Agresti A Caffo B. Multinomial logit random effects models. Stat Model. (2001) 1:81–102. 10.1177/1471082X0100100201

[33] Xiao D Qu Y Huang L Wang Y Li X Mu D. Association between maternal overweight or obesity and cerebral palsy in children: a meta-analysis. PLoS One. (2018) 13:e0205733. 10.1371/journal.pone.0205733 30325944 PMC6191132

[34] Wu YW Colford JM Jr. Chorioamnionitis as a risk factor for cerebral palsy: a meta-analysis. JAMA. (2000) 284:1417–24. 10.1001/jama.284.11.1417 10989405

[35] Zeng Y Tang Y Tang J Shi J Zhang L Zhu T . Association between the different duration of breastfeeding and attention deficit/hyperactivity disorder in children: a systematic review and meta-analysis. Nutr Neurosci. (2018) 23:811–23. 10.1080/1028415X.2018.1560905 30577717

[36] Gou X Yang L Pan L Xiao D. Association between bronchopulmonary dysplasia and cerebral palsy in children: a meta-analysis. BMJ Open. (2018) 8:e020735. 10.1136/bmjopen-2017-020735 30232102 PMC6150141

[37] Stoneham SM Milne KM Nuttal E Frew GH Sturrock BR Sivaloganathan H . Thrombotic risk in COVID-19: a case series and case-control study. Clin Med. (2020) 20:e76–81. 10.7861/clinmed.2020-0228 32423903 PMC7385762

[38] Bhayana R Som A Li MD Carey DE Anderson MA Blake MA . Abdominal imaging findings in COVID-19: preliminary observations. Radiology. (2020) 297:E207–15. 10.1148/radiol.2020201908 32391742 PMC7508000

[39] Alwafi H Naser AY Qanash S Brinji AS Ghazawi MA Alotaibi B . Predictors of length of hospital stay, mortality, and outcomes among hospitalised COVID-19 patients in Saudi Arabia: a cross-sectional study. J Multidiscip Healthc. (2021) 14:839–52. 10.2147/JMDH.S304788 33883900 PMC8055273

[40] Contou D Pajot O Cally R Logre E Fraissé M Mentec H . Pulmonary embolism or thrombosis in ARDS COVID-19 patients: A French monocenter retrospective study. PLoS One. (2020) 15:e0238413. 10.1371/journal.pone.0238413 32853267 PMC7451560

[41] Leonard-Lorant I Delabranche X Severac F Helms J Pauzet C Collange O . Acute pulmonary embolism in COVID-19 patients on CT angiography and relationship to D-Dimer levels. Radiology. (2020) 296:E189–91. 10.1148/radiol.2020201561 32324102 PMC7233397

[42] Jain R Young M Dogra S Kennedy H Nguyen V Jones S . COVID-19 related neuroimaging findings: a signal of thromboembolic complications and a strong prognostic marker of poor patient outcome. J Neurol Sci. (2020) 414:116923. 10.1016/j.jns.2020.116923 32447193 PMC7236667

[43] Galloway JB Norton S Barker RD Brookes A Carey I Clarke BD . A clinical risk score to identify patients with COVID-19 at high risk of critical care admission or death: an observational cohort study. J Infect. (2020) 81:282–8. 10.2139/ssrn.3590486 32479771 PMC7258846

[44] Avruscio G Camporese G Campello E Bernardi E Persona P Passarella C . COVID-19 and venous thromboembolism in intensive care or medical ward. Clin Transl Sci. (2020) 13:1108–14. 10.1111/cts.12907 32989908 PMC7567296

[45] Abizaid A Campos CM Guimarães PO Costa JR Jr Falcão BAA Cavalcante R . Patients with COVID-19 who experience a myocardial infarction have complex coronary morphology and high in-hospital mortality: primary results of a nationwide angiographic study. Catheter Cardiovasc Interv. (2021) 98:E370–e8. 10.1002/ccd.29709 33904638 PMC8239511

[46] Alharthy A Aletreby W Faqihi F Balhamar A Alaklobi F Alanezi K . Clinical characteristics and predictors of 28-day mortality in 352 critically ill patients with COVID-19: a retrospective study. J Epidemiol Glob Health. (2021) 11:98–104. 10.2991/jegh.k.200928.001 33095982 PMC7958266

[47] Anderson JJ Ho FK Niedzwiedz CL Katikireddi SV Celis-Morales C Iliodromiti S . Remote history of VTE is associated with severe COVID-19 in middle and older age: UK Biobank cohort study. J Thromb Haemost. (2021) 19:2533–8. 10.1111/jth.15452 34242477 PMC8420476

[48] Arribalzaga K Martínez-Alfonzo I Díaz-Aizpún C Gutiérrez-Jomarrón I Rodríguez M Castro Quismondo N . Incidence and clinical profile of venous thromboembolism in hospitalized COVID-19 patients from Madrid region. Thromb Res. (2021) 203:93–100. 10.1016/j.thromres.2021.05.001 33989984 PMC8106233

[49] Fournier M Faille D Dossier A Mageau A Nicaise Roland P Ajzenberg N . Arterial Thrombotic events in adult inpatients with COVID-19. Mayo Clin Proc. (2021) 96:295–303. 10.1016/j.mayocp.2020.11.018 33549252 PMC7691140

[50] Purroy F Arqué G. Influence of thromboembolic events in the prognosis of COVID-19 hospitalized patients. results from a cross sectional study. PLoS One. (2021) 16:e0252351. 10.1371/journal.pone.0252351 34106984 PMC8189499

[51] Riyahi S Dev H Behzadi A Kim J Attari H Raza SI . Pulmonary embolism in hospitalized patients with COVID-19: a multicenter study. Radiology. (2021) 301:E426–33. 10.1148/radiol.2021210777 34254850 PMC8294351

[52] Scudiero F Silverio A Di Maio M Russo V Citro R Personeni D . Pulmonary embolism in COVID-19 patients: prevalence, predictors and clinical outcome. Thromb Res. (2021) 198:34–9. 10.1016/j.thromres.2020.11.017 33271421 PMC7669475

[53] Violi F Ceccarelli G Cangemi R Cipollone F D'Ardes D Oliva A . Arterial and venous thrombosis in coronavirus 2019 disease (Covid-19): relationship with mortality. Intern Emerg Med. (2021) 16:1231–7. 10.1007/s11739-020-02621-8 34218413 PMC8255055

[54] Wang W Sun Q Bao Y Liang M Meng Q Chen H . Analysis of risk factors for thromboembolic events in 88 patients with COVID-19 pneumonia in Wuhan, China: a retrospective descriptive report. Med Sci Monit. (2021) 27:e929708. 10.12659/MSM.929708 33839733 PMC8047776

[55] Paz Rios LH Minga I Kwak E Najib A Aller A Lees E . Prognostic value of venous thromboembolism risk assessment models in patients with severe COVID-19. TH Open. (2021) 5:e211–9. 10.1055/s-0041-1730293 34179684 PMC8219405

[56] Duarte-Neto AN Monteiro RAA da Silva LFF Malheiros DMAC de Oliveira EP Theodoro-Filho J . Pulmonary and systemic involvement of COVID-19 assessed by ultrasound-guided minimally invasive autopsy. Histopathology. (2020) 77:186–97. 10.1111/his.14160 32443177 PMC7280721

[57] Dolhnikoff M Duarte-Neto AN de Almeida Monteiro RA da Silva LFF de Oliveira EP Saldiva PHN . Pathological evidence of pulmonary thrombotic phenomena in severe COVID-19. J Thromb Haemost. (2020) 18:1517–9. 10.1111/jth.14844 32294295 PMC7262093

[58] Giannis D Ziogas IA Gianni P. Coagulation disorders in coronavirus infected patients: COVID-19, SARS-CoV-1, MERS-CoV and lessons from the past. J Clin Virol. (2020) 127:104362. 10.1016/j.jcv.2020.104362 32305883 PMC7195278

[59] Helms J Tacquard C Severac F Leonard-Lorant I Ohana M Delabranche X . High risk of thrombosis in patients with severe SARS-CoV-2 infection: a multicenter prospective cohort study. Intensive Care Med. (2020) 46:1089–98. 10.1007/s00134-020-06062-x 32367170 PMC7197634

[60] Amasamy R Milne KM Stoneham SM Chevassut TJ. Molecular mechanisms for thrombosis risk in black people: a role in excess mortality from Covid-19. Br J Haematol. (2020) 190:e78–80. 10.1111/bjh.16869 32438458 PMC7280579

[61] Wu J Zhang J Sun X Wang L Xu Y Zhang Y . Influence of diabetes mellitus on the severity and fatality of SARS-CoV-2 infection. Diabetes Obes Metab. (2020) 22:1907–14. 10.1111/dom.14105 32496012 PMC7300679

[62] Xie XD Chen JZ Wang XX Zhang FR Liu YR. Age- and gender-related difference of ACE2 expression in rat lung. Life Sci. (2006) 78:2166–71. 10.1016/j.lfs.2005.09.038 16303146 PMC7094566

[63] La Vignera S Cannarella R Condorelli RA Torre F Aversa A Calogero AE. Sex-specific SARS-CoV-2 mortality: among hormone-modulated ACE2 expression, risk of venous thromboembolism and hypovitaminosis D. Int J Mol Sci. (2020) 21:2948. 10.3390/ijms21082948 32331343 PMC7215653

[64] Kuba K Imai Y Penninger JM. Angiotensin-converting enzyme 2 in lung diseases. Curr Opin Pharmacol. (2006) 6:271–6. 10.1016/j.coph.2006.03.001 16581295 PMC7106490

[65] Guzik TJ Mohiddin SA Dimarco A Patel V Savvatis K Marelli-Berg FM . COVID-19 and the cardiovascular system: implications for risk assessment, diagnosis, treatment options. Cardiovasc Res. (2020) 116:1666–87. 10.1093/cvr/cvaa106 32352535 PMC7197627

[66] Gemmati D Bramanti B Serino ML Secchiero P Zauli G Tisato V. COVID-19 and individual genetic susceptibility/receptivity: role of ACE1/ACE2 genes, immunity, inflammation and coagulation. might the double X-chromosome in females be protective against SARS-CoV-2 compared to the single X-Chromosome in males? Int J Mol Sci. (2020) 21:3474. 10.3390/ijms21103474 32423094 PMC7278991

[67] Albini A Di Guardo G Noonan DM Lombardo M. The SARS-CoV-2 receptor, ACE-2, is expressed on many different cell types: implications for ACE-inhibitor- and angiotensin II receptor blocker-based cardiovascular therapies. Intern Emerg Med. (2020) 15:759–66. 10.1007/s11739-020-02364-6 32430651 PMC7236433

