# Supplementary material for: Cumulative Evidence for the Association of Thrombosis and the Prognosis of COVID-19: Systematic Review and Meta-Analysis
Source: Front Cardiovasc Med. 2022 Jan 25;8:819318. doi: 10.3389/fcvm.2021.819318 (PMC8821518; doi:10.3389/fcvm.2021.819318)
Supplement: Supplementary file 2 [file Table_2.DOCX]

**Newcastle - Ottawa Quality Assessment Scale results for cohort and cross-sectional studies**

|  | **Selection** | | | | **Comparability** | **Outcome** | | |  |
| --- | --- | --- | --- | --- | --- | --- | --- | --- | --- |
| **Study** | **Representativeness of the Exposed Cohort** | **Selection of the Non-Exposed Cohort** | **Ascertainment of Exposure** | **Demonstration That Outcome of Interest Was Not Present at Start of Study** | **Comparability of Cohorts on the Basis of the Design or Analysis** | **Assessment of Outcome** | **Was Follow-Up Long Enough for Outcomes to Occur** | **Adequacy of Follow Up of Cohorts** | **Score** |
| Zhang 2020 | ***** | ***** | ***** | ***** | ***** | ***** | ***** | **/** | **7** |
| Yaghi, Shadi. 2020 | ***** | ***** | ***** | ***** | ***** | ***** | ***** | **/** | **7** |
| Stoneham, Simon M.2020 | ***** | ***** | ***** | **/** | ***** | ***** | ***** | **/** | **6** |
| Middeldorp, S.2020 | ***** | ***** | ***** | ***** | ***** | ***** | ***** | **/** | **7** |
| Leonard-Lorant, Ian2020 | ***** | ***** | ***** | ***** | ***** | ***** | ***** | **/** | **7** |
| Klok, F. A.2020 | ***** | ***** | ***** | ***** | ***** | ***** | ***** | **/** | **7** |
| Jain, R.2020 | ***** | ***** | ***** | ***** | ***** | ***** | ***** | ***** | **8** |
| Bhayana, R.2020 | ***** | ***** | ***** | **/** | ***** | ***** | ***** | **/** | **6** |
| Ren, B.2020 | ***** | ***** | ***** | ***** | ***** | ***** | ***** | **/** | **7** |
| Galloway, James B 2020 | ***** | ***** | ***** | ***** | ***** | ***** | ***** | **/** | **7** |
| Corrado Lodigiani 2020 | ***** | ***** | ***** | **/** | ***** | ***** | ***** | **/** | **6** |
| Avruscio 2020 | ***** | ***** | ***** | ***** | ***** | ***** | ***** | **/** | **7** |
| Contou 2020 | ***** | ***** | ***** | **/** | ***** | ***** | ***** | **/** | **6** |
| Abizaid 2021 | ***** | ***** | ***** | ***** | ***** | ***** | ***** | **/** | **7** |
| Alharthy 2021 | ***** | ***** | ***** | ***** | ***** | ***** | ***** | **/** | **7** |
| Alwafi 2021 | ***** | ***** | ***** | ***** | ***** | ***** | ***** | ***** | **8** |
| Anderson 2021 | ***** | ***** | ***** | ***** | ***** | ***** | ***** | ***** | **8** |
| Arribalzaga 2021 | ***** | ***** | ***** | ***** | ***** | ***** | ***** | ***** | **8** |
| Fournier 2021 | ***** | ***** | ***** | ***** | ***** | ***** | ***** | ***** | **8** |
| Purroy 2021 | ***** | ***** | ***** | ***** | ***** | ***** | ***** | ***** | **8** |
| Riyahi 2021 | ***** | ***** | ***** | **/** | ***** | ***** | ***** | **/** | **6** |
| Scudiero 2021 | ***** | ***** | ***** | ***** | ***** | ***** | ***** | **/** | **7** |
| Violi 2021 | ***** | ***** | ***** | ***** | ***** | ***** | ***** | ***** | **8** |
| Wang 2021 | ***** | ***** | ***** | **/** | ***** | ***** | ***** | **/** | **6** |
| Paz Rios 2021 | ***** | ***** | ***** | ***** | ***** | ***** | ***** | **/** | **7** |
