# Supplementary material for: Cumulative Evidence for the Association of Thrombosis and the Prognosis of COVID-19: Systematic Review and Meta-Analysis
Source: Front Cardiovasc Med. 2022 Jan 25;8:819318. doi: 10.3389/fcvm.2021.819318 (PMC8821518; doi:10.3389/fcvm.2021.819318)
Supplement: Supplementary file 3 [file Table_3.DOCX]

MEDLINE Ovid 1624

1. exp Thrombosis/pc

2. exp Embolism/pc

3. (thrombosis or thrombotic or thrombus or thrombi or thromboembol*).tw.

4. (emboli* or embolus).tw.

5. clot?.tw.

6. (DVT or VTE or PE).tw.

7. or/1‐6

8. SARS-CoV-2.mp.

9. COVID-19.mp.

10. or/8-9

11. 7 and 10

12. (risk or mortalit: or cohort).tw.

13. 12 and 13

Embase Ovid 1965

1. exp thrombosis/

2. exp embolism/

3. (thrombosis or thrombotic or thrombus or thrombi or thromboembol*).tw.

4. (emboli* or embolus).tw.

5. (phlebothrombo* or phlebitis).tw.

6. exp blood clotting/

7. clot.tw.

8. (DVT or VTE or PE).ti,ab.

9. or/1‐8

10.(SARS-CoV-2).ti,ab.

11.(COVID-19).ti,ab.

12. or/10-11

13. (risk or mortalit: or cohort).tw.

14. 9 and 12 and 13

15. limit 14 to (embase and english and article)

Web of Science 3095

#1 TS=(thrombosis or thrombotic or thrombus or thrombi or thromboembol* OR emboli* OR embolus OR phlebothrombo* or phlebitis OR clot OR DVT OR VTE OR PE)

#2 TS=(SARS-CoV-2 OR COVID-19)

#3 #1 AND #2

#4 TS=(risk or mortalit: or cohort)

#5 #3 AND #4

1965+1624+3095+related 2=6686
