# Supplementary material for: Cumulative Evidence for the Association of Thrombosis and the Prognosis of COVID-19: Systematic Review and Meta-Analysis
Source: Front Cardiovasc Med. 2022 Jan 25;8:819318. doi: 10.3389/fcvm.2021.819318 (PMC8821518; doi:10.3389/fcvm.2021.819318)

Tests for Publication Bias

**Association between thrombosis and mortality**

**Begg's Test**

adj. Kendall's Score (P-Q) = 53

Std. Dev. of Score = 28.58

Number of Studies = 19

z = 1.85

Pr > |z| = 0.064

z = 1.82 (continuity corrected)

Pr > |z| = 0.069 (continuity corrected)

**Egger's test**

------------------------------------------------------------------------------

Std_Eff | Coef. Std. Err. t P>|t| [95% Conf. Interval]

-------------+----------------------------------------------------------------

slope | .947036 .1409419 6.72 0.000 .6496746 1.244397

bias | -.1183216 .8036464 -0.15 0.885 -1.813867 1.577224


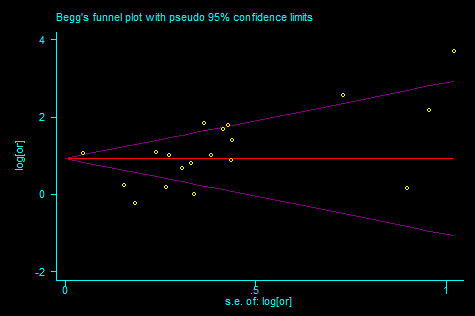


**Association between thrombosis and critical status**

**Begg's Test**

adj. Kendall's Score (P-Q) = 3

Std. Dev. of Score = 11.18

Number of Studies = 10

z = 0.27

Pr > |z| = 0.788

z = 0.18 (continuity corrected)

Pr > |z| = 0.858 (continuity corrected)

Egger's test

------------------------------------------------------------------------------

Std_Eff | Coef. Std. Err. t P>|t| [95% Conf. Interval]

-------------+----------------------------------------------------------------

slope | -.0386543 .3883558 -0.10 0.923 -.9342045 .8568959

bias | 2.271611 1.115404 2.04 0.076 -.300516 4.843738


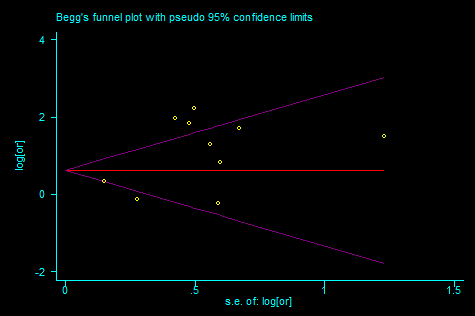

Supplement: Supplementary file 4 [file Table_4.DOCX]
